# A 21-Year-Old Male With Metastatic Colorectal Cancer: A Case Report and Literature Overview of Early-Onset Colorectal Cancer

**DOI:** 10.7759/cureus.76675

**Published:** 2024-12-31

**Authors:** Daniel Aintabi, Stephanie Mackenzie, Wael Al-Yaman, Kevin Wenzke, Jeffrey Berinstein

**Affiliations:** 1 Internal Medicine, Trinity Health Ann Arbor, Ann Arbor, USA; 2 Gastroenterology and Hepatology, Cleveland Clinic Foundation, Cleveland, USA; 3 Gastroenterology and Hepatology, Huron Gastro, Ann Arbor, USA; 4 Gastroenterology and Hepatology, University of Michigan, Ann Arbor, USA

**Keywords:** chemotherapy for advanced colorectal cancer, colorectal cancer, colorectal cancer liver metastases, metastatic colorectal cancer (mcrc), young onset colorectal cancer

## Abstract

Early-onset colorectal cancer (CRC) has been on the rise since the start of the twenty-first century. While the etiology behind this increase remains unclear, the United States Preventive Services Task Force (USPSTF) has decreased the recommended age to begin screening for CRC to 45 years. This case report reviews the literature on CRC in the young population while presenting a case of a 21-year-old male with early-onset metastatic colorectal cancer without a hereditary etiology.

## Introduction

In 2021, the US Preventive Services Task Force (USPSTF) recommended that adults aged 45-49 begin colorectal cancer (CRC) screening as a category B recommendation. These changes to prior recommendations were based on a reported 15% increase in the incidence of CRC in adults aged 40-49 years from 2000-2002 to 2014-2016 [1]. With a reported 5-35% of early-onset CRC cases attributed to hereditary syndromes such as Lynch or polyposis syndromes, the remaining percentage indicates that the majority of early-onset CRC patients lack predisposing genetic conditions [2,3]. While the reasoning behind the increasing incidence of early-onset CRC remains unclear, evidence supports the hypothesis that increases in modifiable risk factors such as obesity, metabolic syndrome, insulin resistance, diet patterns, tobacco use, and alcohol consumption are contributory [4-6]. This case involves a 21-year-old male who initially presented with abdominal pain and multiple liver lesions and was subsequently diagnosed with metastatic colorectal cancer.

This case report was presented as a poster presentation at the American College of Physicians Internal Medicine Meeting in San Diego, April 28, 2023.

## Case presentation

**Figure 1:**
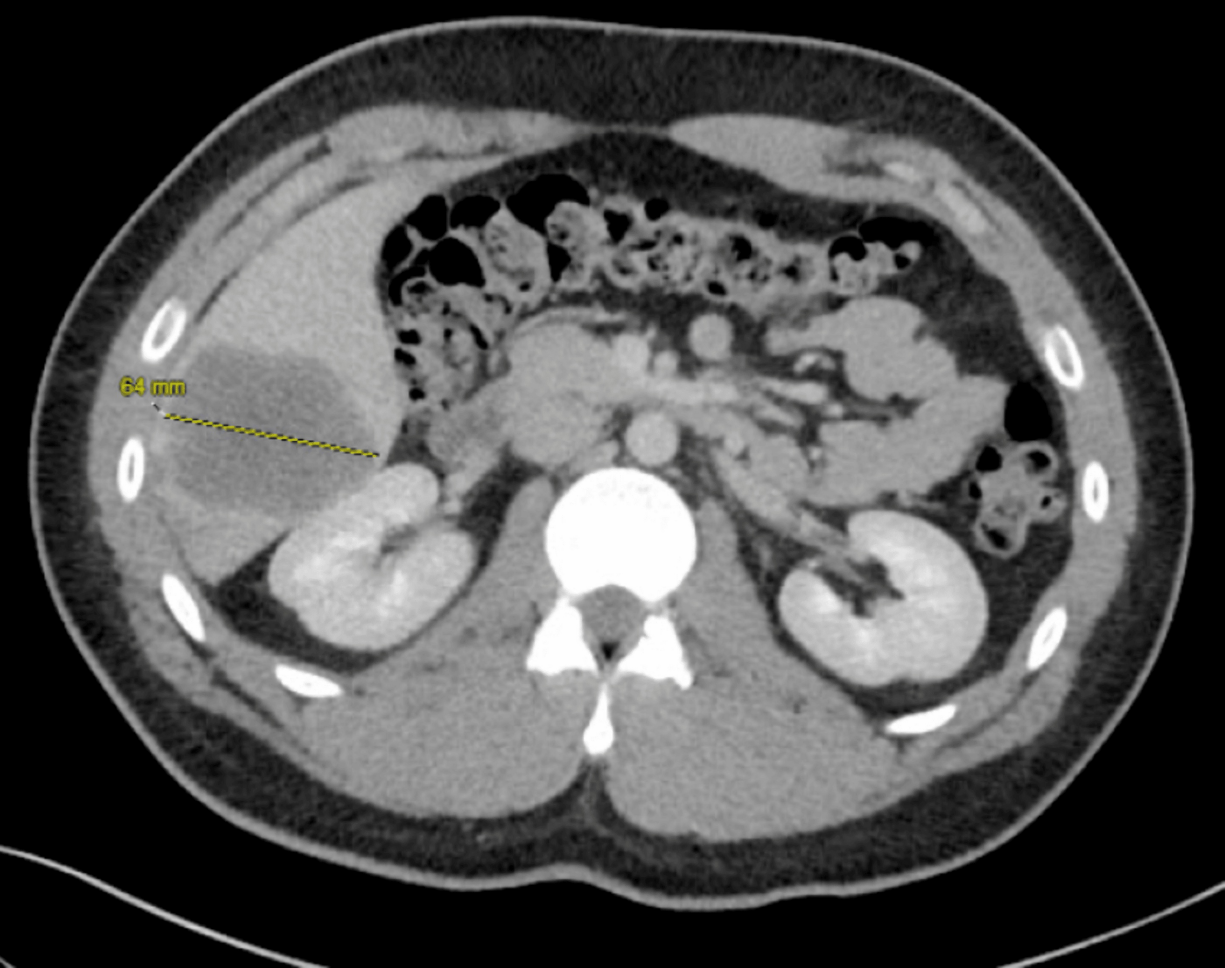
CT abdomen and pelvis (AP) demonstrating the largest liver metastasis, measuring 6.4 cm.

**Figure 2:**
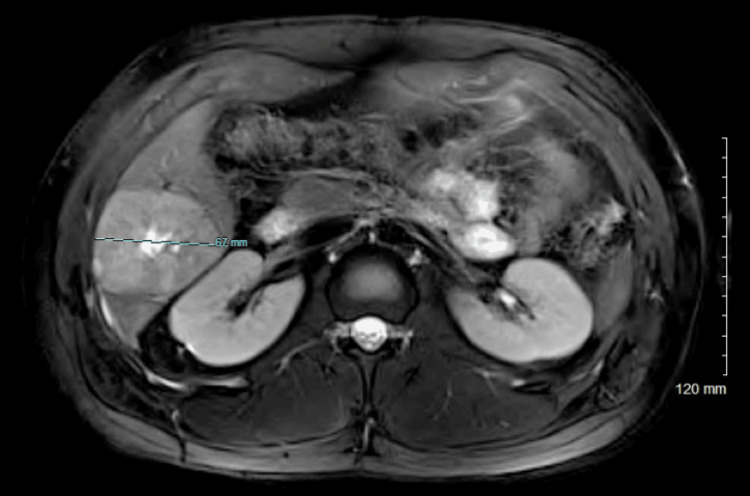
MR abdomen showing the largest hepatic metastasis.

A 21-year-old Hispanic male with minimal significant past medical history presented with a one-day history of right upper quadrant pain, subjective fevers, and chills. He denied a personal or family history of inflammatory bowel disease, CRC, or genetic diseases, including Lynch syndrome or familial adenomatous polyposis. His BMI on initial presentation was 31 kg/m². The lipid profile showed triglycerides and cholesterol within normal limits, and fasting glucose was 92 mg/dL; however, hemoglobin A1C was not obtained. A complete blood count (CBC) and comprehensive metabolic panel (CMP) were notable for leukocytosis of 13.7 K/µL. A CT of the chest, abdomen, and pelvis revealed multiple low-attenuation liver lesions concerning for hepatic abscesses versus metastases (Figure 1). The CT also noted fluid collection adjacent to a diverticulum of the sigmoid colon concerning for diverticulitis versus abscess, as well as mild lymphadenopathy in the porta hepatis region. An MR abdomen liver protocol was more reflective of metastases with primary adenocarcinoma in the distal descending/proximal sigmoid colon, as well as an enlarged portacaval lymph node suspicious for metastatic disease (Figure 2). Tumor markers were notable for an elevated carcinoembryonic antigen (CEA) of 73.9 ng/mL (reference: 0-30 ng/mL).

**Figure 3:**
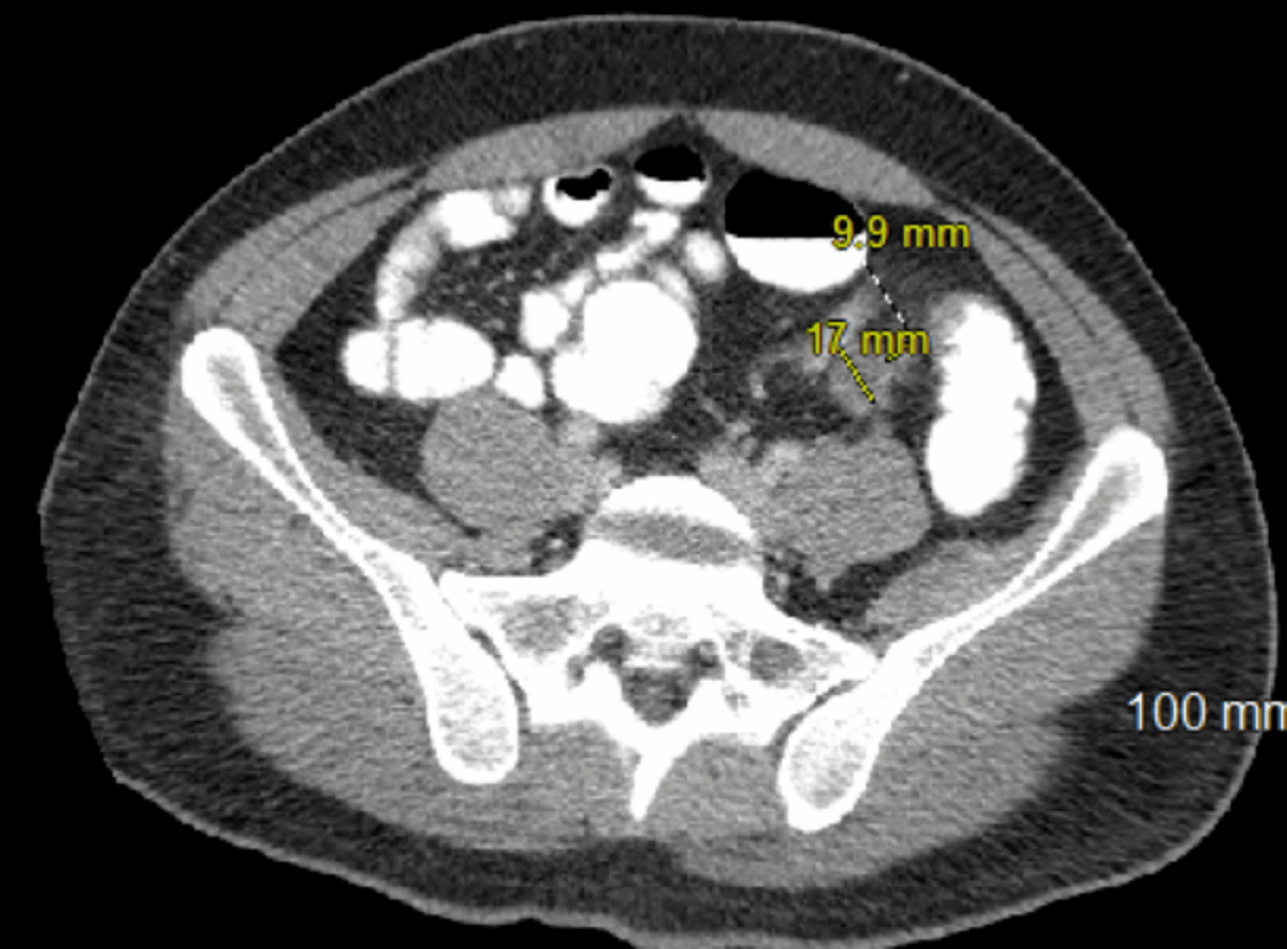
CT abdomen and pelvis (AP) with rectal contrast demonstrating two colonic apple core lesions measuring 1.7 cm and 1.0 cm.

**Figure 4:**
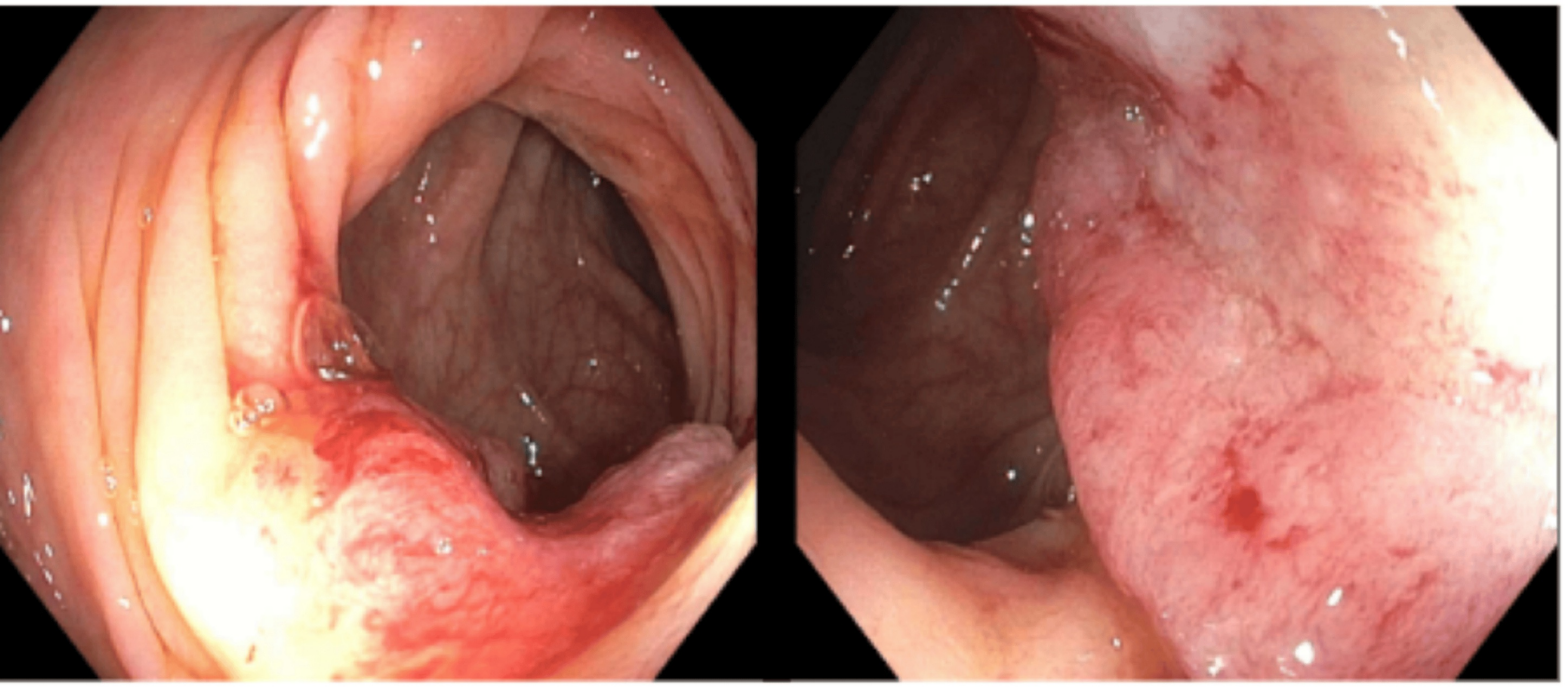
Colonoscopy with ulcerated nonobstructing mass in sigmoid colon.

**Figure 5:**
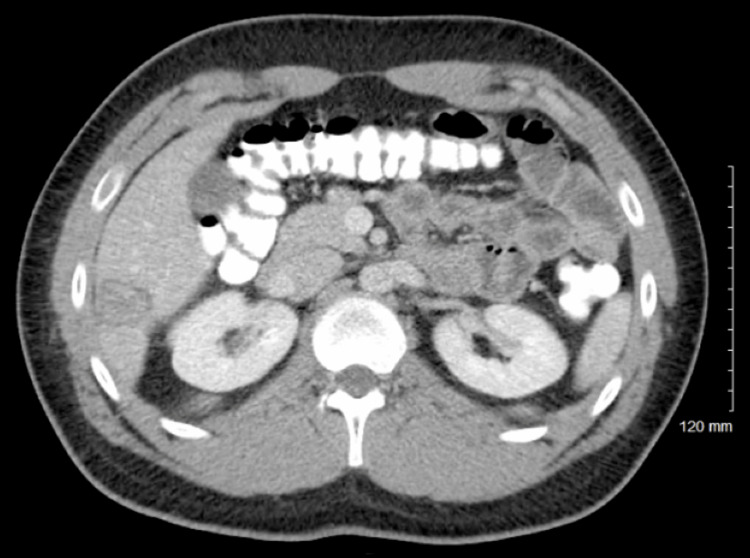
CT after 10 months of chemotherapy and immunotherapy with decreased size of hepatic metastases with the largest measuring 2.9 cm.

Repeat CT abdomen and pelvis with rectal contrast redemonstrated liver lesions and porta hepatis lymphadenopathy, as well as two apple core lesions at the junction of the descending colon and sigmoid colon (Figure 3). On day three of hospitalization, the patient underwent an ultrasound-guided liver biopsy, which was significant for metastatic pleomorphic non-small cell carcinoma with extensive necrosis. On day four, the patient underwent esophagogastroduodenoscopy (EGD), which was unremarkable, followed by colonoscopy demonstrating an ulcerated, nonobstructing 3 cm mass in the sigmoid colon, with biopsy positive for invasive adenocarcinoma (Figure 4). Immunohistochemical stains from the endoscopic biopsy of the primary tumor for MLH1, MSH2, MSH6, and PMS2 showed intact nuclear staining in the neoplasm and background control tissue, consistent with intact mismatch repair protein expression. Genomic testing from the liver biopsy revealed CKKN2A/B loss, PIK3CA, MTAP, and SMAB4 mutations, wild-type RAS/RAF, and a tumor mutational burden (TMB) of 8. A multidisciplinary team consisting of gastroenterology, hepatology, oncology, palliative care, and colorectal surgery recommended initiating chemotherapy following fertility preservation. Initiation of chemotherapy with FOLFOX and immunotherapy with panitumumab was delayed by two months due to marantic endocarditis and the onset of bilateral subsegmental pulmonary emboli. He was initially started on Eliquis; however, with subsequent progression of PE despite therapy, he was switched to Lovenox. The patient was advised to instruct his family members to discuss screening options with their providers. He has since had a positive response to chemotherapy and immunotherapy with a decreased size of multiple hepatic metastases (Figure 5). He is currently three years out from his initial diagnosis, following up with medical oncology and receiving maintenance chemotherapy with capecitabine.

## Discussion

The incidence of CRC in young adults has been increasing over the last 20 years [1]. This case demonstrates the importance of physician awareness in identifying concerning signs and symptoms of CRC in the younger age population. The most common presenting symptoms of early-onset CRC are anemia, hematochezia, abdominal pain, and decreased appetite [7]. Diseases with a predisposition for early-onset CRC include inflammatory bowel disease, nonfamilial adenomatous polyposis (Lynch syndrome), and familial adenomatous polyposis, with screening guidelines in these populations implemented for earlier detection of CRC [8-11]. This case consisted of a young patient without a family predisposition to hereditary etiology for colorectal cancer, with immunohistochemical staining suggesting against Lynch disease. It is unique in that it identifies the importance of consideration for causes of sporadic early-onset colorectal cancer.

Given the increasing rates of CRC in younger populations, it is important to identify modifiable risk factors. Tobacco use and sedentary lifestyles are both associated with early-onset CRC [4,5]. Recent studies have also attributed increasing rates of CRC in younger populations to increasing early-onset obesity and type II diabetes mellitus [2]. The link between obesity and colorectal cancer has been hypothesized to be related to the proinflammatory state, unfavorable adipokine profile, macrophage recruitment, and oxidative stress associated with obesity [6]. Similarly, as part of the metabolic syndrome associated with obesity, insulin resistance has been linked to increased cases of CRC. It is postulated that this increase is secondary to the proliferative and apoptotic effects of insulin and insulin-like growth factor pathways such as IGF1 pathways [12-14]. In a meta-analysis of over 30,000 cases of early-onset CRC and 10 million controls, type 2 diabetes mellitus was associated with a significantly increased risk of CRC, with a pooled odds ratio of 1.43 (95% CI 1.08-1.8) [15]. Various dietary factors associated with the Western diet, such as higher amounts of indigestible carbohydrates and N-nitroso compounds from processed meats, have also been associated with a higher risk of CRC development [16].

CRC is associated with an increased risk of thrombotic events secondary to the hypercoagulable state of malignancy. This patient presented with metastatic CRC with evidence of both marantic endocarditis and pulmonary emboli. This is an important complication of CRC to highlight, given that patients with CRC have an increased risk of thrombotic events, especially within the first six months of diagnosis [17]. Also demonstrated in this case is the importance of fertility preservation in patients with early-onset CRC prior to initiation of chemotherapy with FOLFOX, due to the risk of chemotherapy-induced infertility. This is particularly significant due to the adverse event profile of oxaliplatin, a chemotherapy agent used in FOLFOX therapy, known to cause infertility through decreased sperm counts in men and premature ovarian failure in women [18]. Fertility preservation, performed prior to initiation of FOLFOX therapy, consists of sperm preservation in men and egg or embryo preservation in women.

While the USPSTF has recommended earlier CRC screening in the average-risk population, the question remains whether the increasing incidence of early-onset colorectal cancer necessitates further screening interventions. This, however, cannot be based on isolated cases of early-onset CRC. With the recent advancements in stool-based testing, one potential consideration would be the implementation of CRC screening using non-colonoscopy screening in younger age populations. Multitargeted stool DNA testing, specifically compared to fecal occult blood test (FOBT) and fecal immunochemical testing (FIT), has been shown to have the highest sensitivity for identifying colorectal cancer [19]. Additionally, multitargeted stool DNA testing was associated with higher rates of adherence compared with other stool-based testing, which is important given that it was shown that only 56% of patients with positive stool-based testing had diagnostic colonoscopy within one year [20,21]. Although stool-based testing has been shown to be a cost-effective strategy compared to no screening in the general population, its cost-effectiveness as a screening tool for early-onset CRC has yet to be studied, posing a potential limitation for its use in younger populations [22].

## Conclusions

This case highlights the importance of physician awareness of increasing CRC rates in younger populations to allow for earlier detection of CRC in patients with concerning signs and symptoms. While the etiology for the rise in early-onset CRC remains unclear, further research is needed to determine risk factors associated with CRC in younger populations. Identifying these risk factors would permit the implementation of appropriate screening guidelines in patients at increased risk for early-onset CRC.
